# Hyperthermia and chemotherapy using Fe(Salen) nanoparticles might impact glioblastoma treatment

**DOI:** 10.1038/srep42783

**Published:** 2017-02-20

**Authors:** Makoto Ohtake, Masanari Umemura, Itaru Sato, Taisuke Akimoto, Kayoko Oda, Akane Nagasako, Jeong-Hwan Kim, Takayuki Fujita, Utako Yokoyama, Tomohiro Nakayama, Yujiro Hoshino, Mai Ishiba, Susumu Tokura, Masakazu Hara, Tomoya Muramoto, Sotoshi Yamada, Takatsugu Masuda, Ichio Aoki, Yasushi Takemura, Hidetoshi Murata, Haruki Eguchi, Nobutaka Kawahara, Yoshihiro Ishikawa

**Affiliations:** 1 Cardiovascular Research Institute, Yokohama City University, Graduate School of Medicine, Yokohama, 236-0004, Japan; 2 Department of Neurosurgery, Yokohama City University, Graduate School of Medicine, Yokohama, 236-0004, Japan; 3 Department of Oral and Maxillofacial Surgery, Yokohama City University, Graduate School of Medicine, Yokohama, 236-0004, Japan; 4 Department of Veterinary Medicine, Nihon University, Fujisawa, 252-8510, Japan; 5 Department of Environment and Natural Sciences, Graduate School of Environment and Information Sciences, Yokohama National University, Yokohama, 240-8501, Japan; 6 Advanced Applied Science Department, Research Laboratory, IHI Corporation, Yokohama, 235-0031, Japan; 7 Heat and Fluid Dynamics Department, Research Laboratory, IHI Corporation, Yokohama, 235-0031, Japan; 8 Collage of Science and Engineering, Kanazawa University, Kakuma, 920-1192, Japan; 9 Neutron Science Laboratory, Institute for Solid State Physics, the University of Tokyo, Shirakata, Tokai, 319-1106, Japan; 10 Department of Molecular Imaging and Theranostics, National Institute of Radiological Sciences, QST, Chiba, 263-8555, Japan; 11 Electrical and Computer Engineering, Yokohama National University, Yokohama, 240-8501, Japan

## Abstract

We previously reported that μ-oxo N,N’-bis(salicylidene)ethylenediamine iron [Fe(Salen)], a magnetic organic compound, has direct anti-tumor activity, and generates heat in an alternating magnetic field (AMF). We showed that Fe(Salen) nanoparticles are useful for combined hyperthermia-chemotherapy of tongue cancer. Here, we have examined the effect of Fe(Salen) on human glioblastoma (GB). Fe(Salen) showed *in vitro* anti-tumor activity towards several human GB cell lines. It inhibited cell proliferation, and its apoptosis-inducing activity was greater than that of clinically used drugs. Fe(Salen) also showed *in vivo* anti-tumor activity in the mouse brain. We evaluated the drug distribution and systemic side effects of intracerebrally injected Fe(Salen) nanoparticles in rats. Further, to examine whether hyperthermia, which was induced by exposing Fe(Salen) nanoparticles to AMF, enhanced the intrinsic anti-tumor effect of Fe(Salen), we used a mouse model grafted with U251 cells on the left leg. Fe(Salen), BCNU, or normal saline was injected into the tumor in the presence or absence of AMF exposure. The combination of Fe(Salen) injection and AMF exposure showed a greater anti-tumor effect than did either Fe(Salen) or BCNU alone. Our results indicate that hyperthermia and chemotherapy with single-drug nanoparticles could be done for GB treatment.

Glioblastoma (GB: WHO Grade IV) is the most common and most aggressive brain tumor in adults. It has a very poor prognosis, because of its invasiveness, its resistance to treatment, and the difficulty of total resection^1^,^2^,^3^. Temozolomide (TMZ) combined with extended focal radiotherapy is considered the standard treatment of GB, but median overall survival of GB is still less than 15 months and has changed little in recent decades^3^,^4^,^5^,^6^. Alternative therapeutic approaches have produced some promising results. For example, wafers of 1,3-bis(2-chlorethyl)-1-nitrosourea; BCNU; carmustine placed directly in the resection cavity following operation significantly improved survival^4^,^7^,^8^,^9^. The treatment with BCNU wafers and TMZ has a significant survival benefit compared to the conventional standard therapy^10^,^11^,^12^,^13^.

There have also been various attempts to use mild hyperthermia to cancer therapy^14^,^15^,^16^. The effectiveness of hyperthermia for cancer has been well-known. Hyperthermia increases the susceptibility of cancer cells to therapeutic agents, however with limited success due to the limitations of available technologies^14^,^15^,^17^,^18^,^19^,^20^. A major technical problem with hyperthermia is the difficulty in heating the target to the desired temperature without damaging the surrounding normal tissues. Recent technical advances; nanoparticles, thermo-monitor, and navigation system, have increased interest in this strategy again^21^,^22^.

For example, intratumoral hyperthermic therapy for GB using magnetic iron-oxide nanoparticles (Fe_3_O_4_), which were directly injected into the tumor and exposed to an alternating magnetic field (AMF) to generate heat, has been investigated^23^,^24^,^25^. In a single-arm Phase II study in two centers, this intratumoral thermotherapy afforded a median overall survival of 23.2 months, compared with 14.6 months reported for conventional therapy, and the side effects were not severe^25^.

We previously reported that μ-oxo N,N’-bis(salicylidene)ethylenediamine iron [Fe(Salen)], which is intrinsically magnetic, exhibits anti-tumor activity^26^. *In vivo,* magnetically guided delivery of Fe(Salen) caused a robust decrease in tumor size, and the accumulation of Fe(Salen) could be visualized by magnetic resonance (MR) imaging. We also demonstrated the feasibility of simultaneous hyperthermia-chemotherapy with Fe(Salen) by using AMF-induced heating, generated as a result of hysteresis loss together with Joule loss^26^. Fe(Salen) nanoparticles had not only hyperthermia-inducing effect in an AMF, but also anti-tumor effect, resulting in stable anti-tumor properties when exposed to AMF^27^. Based on these results, we hypothesized that this strategy would also be applicable to treat GB.

Therefore, in this study, we evaluated the anti-tumor and hyperthermia-inducing effects of Fe(Salen) in human GB, both *in vitro* and *in vivo*. The combination of Fe(Salen) injection and AMF exposure (combined hyperthermia-chemotherapy) showed a greater anti-tumor effect in a mouse leg tumor model of GB than did either Fe(Salen) or BCNU alone. Our findings indicate that hyperthermia and chemotherapy with single-drug nanoparticles could be done for GB treatment.

## Materials and Methods

### Reagents and cell culture

μ-oxo N,N’-bis(salicylidene)ethylenediamine iron, Fe(Salen) was purchased from Tokyo Chemical Industry Co. Ltd. It was sonicated for 30 minutes and suspended in normal saline before use, as previously described^26^,^27^. Temozolomide (TMZ) and carmustine (1,3-bis(2-chlorethyl)-1-nitrosourea; BCNU) were purchased from Sigma. Human GB cell lines, U251 (U251MG-Luc, JCRB1386) and YKG (YKG-1, JCRB0746)^28^, were purchased from Japanese Collection of Research Bioresources (JCRB) Cell Bank. U251 cell line has been engineered to express the firefly luciferase gene. Human GB cell line U87 (U87MG, HTB-14) was purchased from American Type Culture Collection (ATCC) (Virginia, USA). Normal human astrocytes (NHA) were purchased from Lonza group, Ltd. In all cases, early-passage cultures were stored and used for experiments. GB cell lines were cultured in Dulbecco’s modified Eagle’s medium (DMEM; Sigma-Aldrich) containing 10% fetal bovine serum (FBS) and 1% penicillin-streptomycin. Normal human astrocytes (NHA) were cultured in specialized medium (AGM BulletKit) purchased from Lonza group, Ltd. D-Luciferin was purchased from Promega (Wisconsin, USA).

### Electric devices

An Alternating magnetic field (AMF) was driven by a transistor inverter (Hot Shot, Ameritherm Inc., New York, USA) and generated by a solenoid copper coil (resistivity: 1.673 × 10^−8^ Ωm) with an inner diameter of 4 cm and outer diameter of 5 cm. Experiments were performed at a frequency of 280 kHz and a current of 335. 4 Arms for 60 minutes^16^,^26^,^27^. Magnetic flux density was simulated with JMAG Designer software, version 14.1 (JSOL Corporation, Tokyo, Japan), employing the 3D finite element method (simulation type: magnetic field analysis (frequency response)). A thermometer (fiber optic thermometer FL-2400, Anritsu Meter Co., Tokyo, Japan) or a thermograph (InfraRed camera, Nippon Avionics Co., Ltd, Tokyo, Japan) was used to determine temperature *in vivo* and *in vitro*^27^.

### Viability analysis

Viability analysis was performed as we previously reported^27^. Calcein-AM and propidium iodide were purchased from Sigma. The influence of AMF for GB cells (U251) and NHA was examined by incubating each cells in 4 cm dishes (5.0 × 10^4^ cells per dish) for 7 days, then applying AMF for 1 hour. At 24 hours after AMF stimulation, surviving cells and dead cells were evaluated by incubation with calcein-AM and propidium iodide, respectively, at 37 °C for 15 minutes. Images were acquired with a fluorescence microscope. Hyperthermia effect was similarly examined. U251 cells and NHA were seeded on each well of 6-well plates (5.0 × 10^4^ cells per well) and incubated for 7 days. Then, U251 cells were incubated at 43 °C and NHA at 43 °C or 47 °C for 30 minutes, 1 hour and 2 hours. Control cells were kept at 37 °C. Surviving cells and dead cells were evaluated at 24 hours after hyperthermia stimulation as above.

### Sodium 2,3,-bis(2-methoxy-4-nitro-5-sulfophenyl)-5-[(phenylamino)-carbonyl]-2H-tetrazolium inner salt (XTT) assay

Cell proliferation assay was performed using a commercial kit, XTT Cell Proliferation Assay Kit (ATCC, Virginia, USA), as previously described^27^,^29^. GB cells were seeded on 96-well plates at 5.0 × 10^3^ cells per well. NHA were seeded at 3.0 × 10^3^ cells per well. Blank control wells contained medium alone without Fe(Salen). Cells were incubated at 37 °C in the presence of Fe(Salen), TMZ or BCNU for 72 hours, then Activated-XTT Solution was added to each well. The plates were returned to the incubator for 2 hours and the absorbance of all wells was measured with a microplate reader.

### Apoptosis assay

Apoptosis assays were performed as previously described^16^,^27^. GB cells were seeded in 6 cm dishes at 1.5 × 10^5^ cells per dish, and incubated for 24 hours. After exposure to Fe(Salen), the cells were incubated at 37 °C for 24 hours. APC Annexin V and 7-amino-actinomycin D (7AAD) (Bio Legend, California, USA) were then added to the tubes. Incubation was continued for 15 minutes at room temperature (25 °C) in darkness, followed by measurement with a fluorescence-activated cell sorter (FACS) Canto^TM^ II (Japan Becton, Dickinson and Company, Tokyo, Japan) within 1 hour.

### Measurement of reactive oxygen species (ROS)

Measurement of ROS was performed as reported^27^,^30^. GB cells were incubated overnight in 96-well plates (1.0 × 10^4^ cells per well) and then exposed to Fe(Salen) nanoparticles at 37 °C for 24 hours. Intracellular ROS was measured using a fluorescent dye, 2′,7′-dichlorofluorescein diacetate (DCFH-DA; Sigma, Japan). ROS production was measured using a microplate reader equipped with a spectrofluorometer (ARVO-Mx, PerkinElmer, Massachusetts, USA) at an emission wavelength of 538 nm and an excitation wavelength of 485 nm.

### Tumor implantation and drug injection

Mice were used for tumor implantation studies, and rats were used for MRI scanning, biodistribution analysis, and toxicity testing because their body size and weight were more suitable for these studies.

Female balb/c nu/nu mice (17–23 g) (Japan SLC, Inc., Shizuoka, Japan), 6 weeks old, were anesthetized with isoflurane together with intraperitoneal injection of tribromoethanol (250 mg/kg). In the case of brain injection, a burr hole was made in the skull 0.5 mm anterior and 2.0 mm lateral to the bregma before tumor cells were stereotactically injected by a 30-gauge injection canula to a depth of 4.0 mm. The injection volume was 10 μl per body^31^,^32^.

Crl: CD (SD) male rats (190–299 g), 8 weeks old, were purchased from Charles River Laboratories International, Inc. (USA). Under general anesthesia, a burr hole was made in the skull 0.2–1.0 mm anterior and 3.0 mm lateral to the bregma, and a canula was inserted to 5.0 mm depth from the outer skull^33^. Fe(Salen) (0.12–0.60 mg/body) was injected into the brain. The injection volume was 10–20 μl per body.

### Evaluation of anti-tumor effects in a mouse brain tumor model

We created a mouse brain tumor model to examine the effect of Fe(Salen) and BCNU. U251 cells expressing luciferase gene were injected stereotactically into the brain (1.0 × 10^6^ cells/body). At 7 days after injection of tumor cells, Fe(Salen) (0.066 mg/body), BCNU (0.021 mg/body), or saline (control) was injected into the same location of the brain (n = 4). Injection volume of each drugs was 10 μl per body. Tumor size was determined from the luciferin-induced photon flux, which was measured once a week for 4 weeks. The maximum photon flux was taken as an indicator of tumor volume. Regression rate was calculated using the following formula:


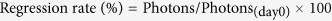


### Evaluation of anti-tumor and hyperthermia effects for GB cells using IVIS *in vitro*

Analysis using an *in vivo imaging system* (IVIS, Xenogen, Alameda, CA, USA)was performed as reported^27^. U251 cells were seeded on 6-well plates (5.0 × 10^4^ cells per well) and incubated for 7 days^27^. Then, Fe(Salen) was added, and the cells were incubated at 43 °C for 1 hour. At 24 hours after hyperthermia, D-luciferin (4.7 mg/well) was added, and after 15 minutes, the bioluminescence signal was examined with IVIS.

### Observation of toxicity

Clinical signs were assessed and recorded twice daily, before and after local Fe(Salen) injection (0.12, 0.60 mg/body) into rat brain (n = 5). Body weight and daily food consumption were calculated 1–2 days and 6–7 days after Fe(Salen) injection. Body weight loss or food consumption decrease of 20% or more compared to the control rats was considered as a potential side effect of treatment.

### Blood chemistry

Toxicity was examined after Fe(Salen) injection (0.60 mg/body) into the brain of rats (n = 5). Serum samples for blood chemistry were obtained by centrifugation of blood samples. Serum ALT (alanine aminotransferase) and AST (aspartate aminotransferase) were measured as parameters of liver function and creatinine as a parameter of renal function, just before Fe(Salen) injection, and at 1 day, 3 days, 7 days, 14 days and 21 days after Fe(Salen) injection.

### Distribution analysis

Whole-body autoradioluminography was performed in order to examine the distribution of ^14^C-Fe(Salen) in the body of rats (n = 1). ^14^C-Fe(Salen) (0.116 MBq/mg; purity 98.69%) was prepared in the laboratories of Yokohama City University Hospital. A single intracerebral injection of ^14^C-Fe(Salen) (0.12 mg/body) was administered to rats, and autoradioluminography was performed 0.25 hours, 24 hours, 168 hours and 504 hours thereafter. ^14^C-Fe(Salen) (0.32 mg/body) was also injected into another site of brain to evaluate intrathecal outflow, and autoradioluminography was performed 3 hours thereafter.

### Magnetic resonance imaging (MRI)

MRI examinations were conducted using conventional T1-weighted imaging (T1WI) (spin-echo sequence, repetition times (TR) = 400 ms, echo times (TE) = 9.6 ms), 3D image (rapid acquisition with relaxation enhancement (RARE) sequence, TR = 400 ms, TE = 26.5 ms, RARE factor = 8), T2-weighted imaging (T2WI) (multi-echo spin-echo sequence, TR = 3000 ms, TE = 40 ms, RARE factor = 8). All examinations were conducted using a 7T magnet (JASTEC-Kobelco, Tokyo, Japan) interfaced to a Bruker console (Bruker BioSpin, Ettlingen, Germany) with a 75-mm-diameter birdcage coil for transmission (Bruker-BioSpin) and a quadrature head coil for reception (Rapid Biomedical, Rimpar, Germany). Following MRI parameters were used: slice thickness = 1.0 mm, matrix = 256 × 256 and field of view (FOV) = 3.2 × 3.2 mm^2^. MRI scans were performed 0.25 hours, 1 hour, 2 hours, 3 hours and 3 days after Fe(Salen) injection into the brain.

### Evaluation of anti-tumor and hyperthermia effects in mouse leg tumor model

We created mouse leg tumor model to examine the effect of Fe(Salen) and BCNU without/with AMF exposure (hyperthermia effect). U251 cells (1.0 × 10^7^ cells/body) were injected into the back of each mouse. When mice carrying U251 cells were injected intraperitoneally with D-luciferin (4.7 mg/body), the tumor emitted a visible light signal that could be monitored using IVIS. The photon flux from the tumor was nearly proportional to the number of light-emitting cells. At 7 days after injection of tumor cells, Fe(Salen) (50 mM), BCNU (50 mM), or saline (control) was injected at the same location. Injection volume was one-third of the tumor volume (n = 6). Some groups were exposed to AMF. Tumor size was measured both manually and in terms of photon flux measured by IVIS for 4 weeks. Manual measurement was performed twice a week. Tumor volume and regression rate were calculated using the following formula^32^:


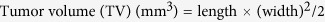



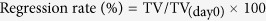


The photon flux was measured once a week for 4 weeks, as described above. Regression rate was calculated using the following formula:


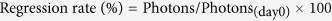


### Ethics statement

Animal experiments were performed according to the Yokohama City University guidelines for experimental animals. The Animal Care and Use Committee at Yokohama City University, School of Medicine, approved all animal studies. All experimental protocols were approved by the Animal Care and Use Committee at Yokohama City University, School of Medicine.

### Date analysis and statistics

Statistical comparisons among groups were performed using Students’ *t*-test or one-factor analysis of variance (ANOVA) with the Bonferroni post hoc test. A *p* value of less than 0.05 was considered statistically significant.

## Results

### Fe(Salen) can generate enough heat to damage GB cells

**Figure 1:**
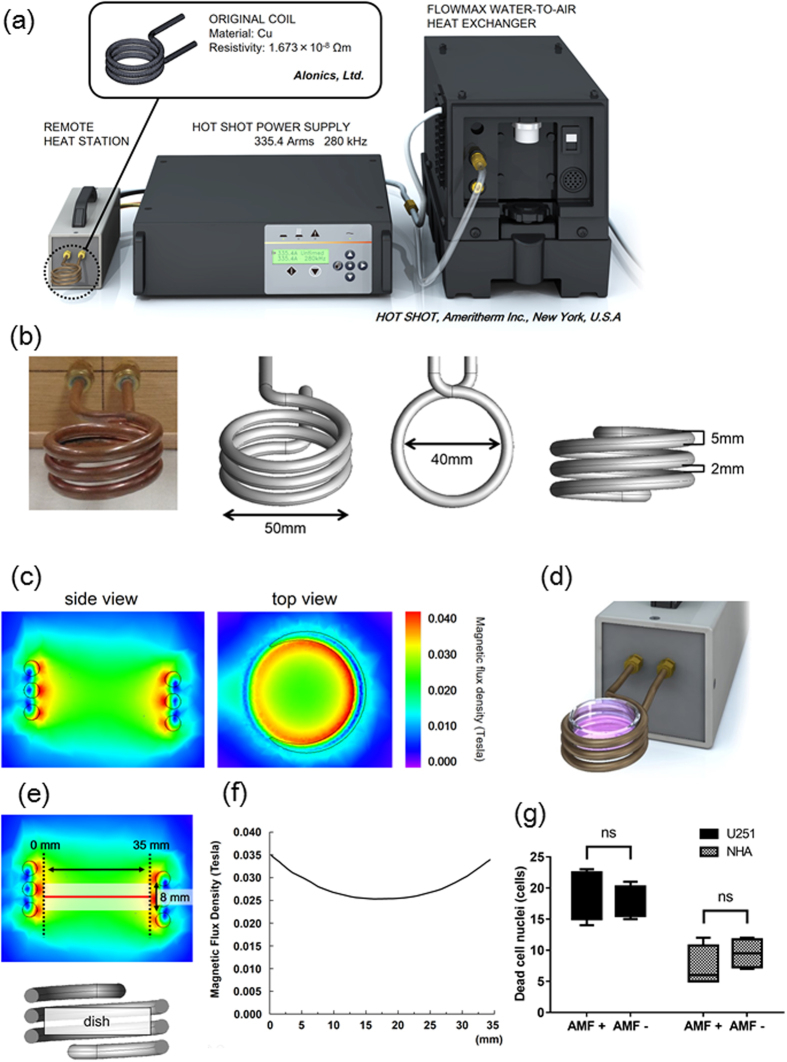
Magnetic flux generated by AMF apparatus does not affect the viability of GB cells and NHA. (**a**) Illustration of the AMF generator. (**b**) Picture and illustrations of the coil of the AMF generator. Arrows indicates inner diameter and outer diameter. (**c**) Magnetic flux density distribution from the simulation (AMF: 280 kHz, 335.4 Arms). Colors indicate values of magnetic flux density (side view and top view). (**d**) Illustration of a culture dish positioned in the coil of the AMF generator. (**e**) Simulation results of the intensity of magnetic flux density due to AMF. (**f**) Relationship between magnetic flux density and distance from the edges of the coil (red line) upon exposure to AMF. (**g**) Ratio of dead GB cells (U251) and dead astrocytes (NHA) with or without AMF exposure (n = 4, ns, not significant).

To examine heat generation by Fe(Salen) exposed to an AMF *in vitro*, we used a commercial AMF generator (Fig. 1a) equipped with a solenoid coil (Fig. 1b). Simulation analysis indicated that the magnetic field was high near the coil (Fig. 1c). The sample dish was placed in the center of the coil (Fig. 1d). Simulation result indicated that the magnetic field was relatively high near the surface of the coil (side view, Fig. 1c). However, the magnetic flux density distribution was reasonably uniform over the dish at this position (red solid line, Fig. 1e and f). We also checked the uniformity of particle size and the magnetism of Fe(Salen) nanoparticles in the suspension (50 mM) with a scanning electron microscope (SEM) and an electron spin resonance (ESR) spectrometer (Supplemental Fig. 1a and b). We performed the X-ray diffraction (XRD) analysis of the Fe(Salen) powder sample (Supplemental Fig. 1c), showing no considerable impurity peaks related to iron oxides. 280 kHz and 335.4 Arms were the maximum parameters of the solenoid coil and AMF device used in this study. Therefore, we adopted AMF parameters that afforded a local temperature of 43 °C (280 kHz and 335.4 Arms) (Supplemental Fig. 1d). Thermography showed that the temperature of Fe(Salen) nanoparticle suspension (50 mM) rose above 43 °C in the AMF (280 kHz and 335.4 Arms) (Supplemental Fig. 1e), and this occurred within 10 minutes (Supplemental Fig. 1f). The vehicle (saline) alone showed that the temperature in the saline solution (blank) rose to 38 °C compared to deionized distilled water when exposed to AMF (Supplemental Fig. 1f). We confirmed that AMF alone did not affect the viability of U251 cells or NHA (Fig. 1g).

### Fe(Salen) shows anti-tumor effect in GB cell lines

**Figure 2:**
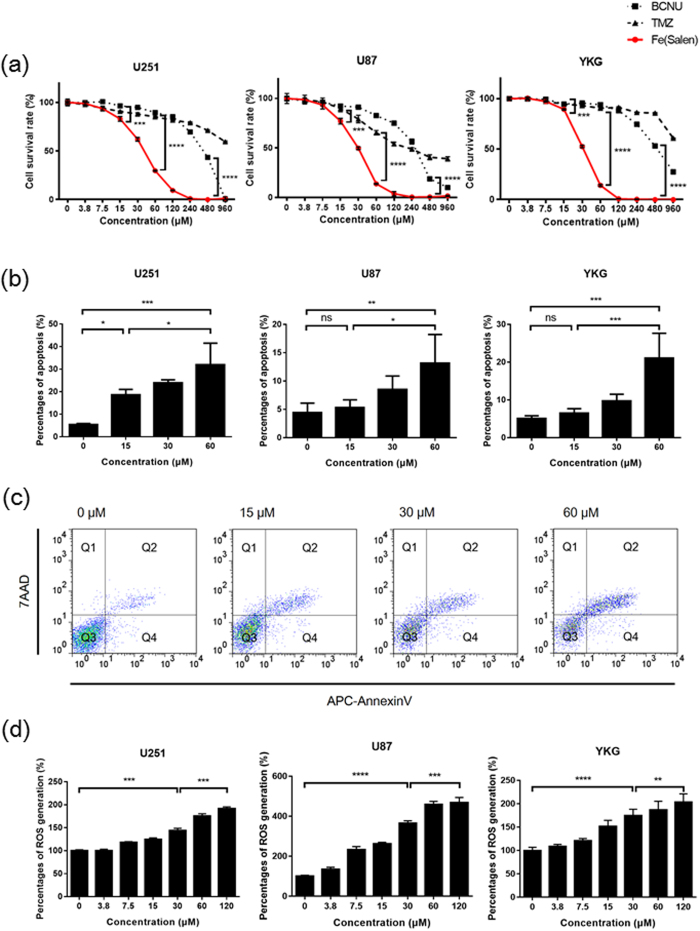
Fe(Salen) nanoparticles inhibit cell proliferation, induce apoptosis and promote ROS generation. (**a**) Effect of Fe(Salen) with saline on proliferation of various tumor cell lines. XTT cell proliferation assays were performed with human GB cell lines U251, U87 and YKG (n = 4, ***p < 0.001, ****p < 0.0001). The IC_50_ values were similar among these cell types and were approximately 30–40 μM. (**b**) Percentages of Fe(Salen)-induced apoptosis in human GB cells (n = 4, ns, not significant, *p < 0.05, **p < 0.01, ***p < 0.001). (**c**) Representative analysis of apoptosis of U251 cells in the presence of Fe(Salen). Early apoptosis and late apoptosis are shown in Q4 and Q2, respectively. (**d**) Effect of Fe(Salen) on ROS production in human GB cells. Fe(Salen) nanoparticles generated ROS in a concentration-dependent manner (n = 4, **p < 0.01, ***p < 0.001, ****p < 0.0001).

We first examined whether Fe(Salen) exhibits anti-tumor activity towards several GB cell lines. Indeed, XTT assay showed strong, dose-dependent anti-tumor effects of Fe(Salen) (Fig. 2a) in agreement with our previous report that Fe(Salen) exhibits cytotoxicity to various cancer cell lines^26^. Indeed, Fe(Salen) had much greater anti-tumor effects than the clinically used drugs TMZ and BCNU. Its IC_50_ values were approximately 30–40 μM among the cell lines examined.

We next examined the pro-apoptotic effect by means of fluorescence-activated cell sorting (FACS). FACS analysis showed that Fe(Salen) dose-dependently induced cell death, early and late apoptosis in all cell lines examined (Fig. 2b and c). We also found that Fe(Salen) dose-dependently generated ROS in several GB cell lines (Fig. 2d), in agreement with our previous findings^26^,^27^. These results indicate that Fe(Salen) is potently cytotoxic to GB cells.

We also examined anti-tumor effect of Fe(Salen) against brain tumor in mice according to the schedule as described in Supplemental Fig. 2a. The mouse model of human GB was made using U251 cells transfected with luciferase-encoding vector. Tumor growth was monitored for 4 weeks with IVIS (Supplemental Fig. 2b). Tumor size in the control group increased slightly, whereas tumor sizes in the Fe(Salen) and the BCNU group were significantly reduced to about 50% after 4 weeks (Supplemental Fig. 2c). The results indicate that Fe(Salen) has anti-tumor effect similar to BCNU *in vivo*.

### Hyperthermia inhibits GB cell proliferation

To evaluate the effect of hyperthermia *per se* on GB cells *in vitro*, we first examined proliferation of U251 cells at 43 °C, at which temperature the tumor growth is known to be suppressed. It has been reported that hyperthermia induces translocation of apoptosis-inducing factor (AIF) and causes apoptosis in human glioma cell lines^34^.

**Figure 3:**
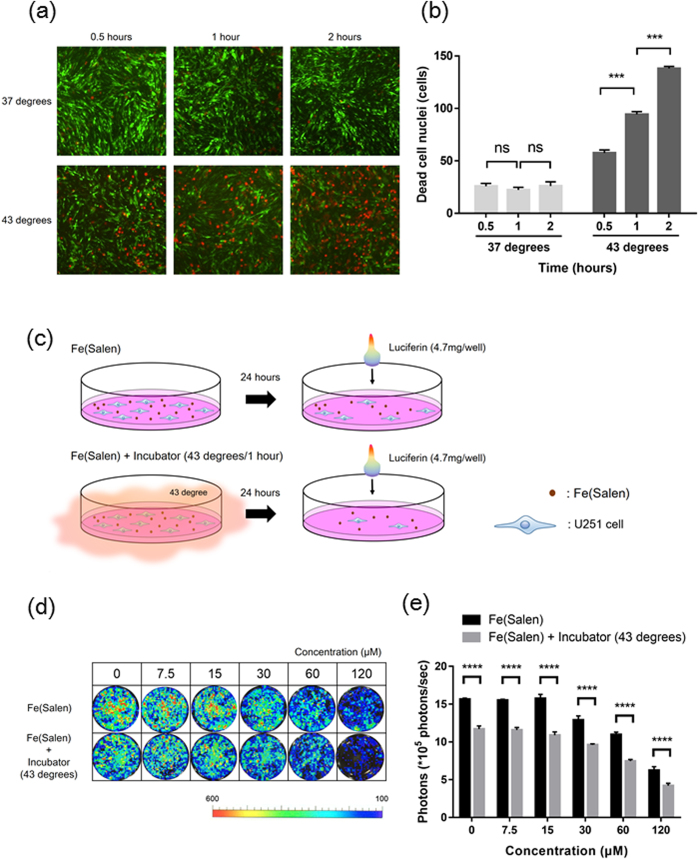
Hyperthermia at 43 °C inhibits cell proliferation of GB *in vitro.* **(a)** Representative fluorescence pictures of calcein-AM and propidium iodide in GB cells (U251) using a fluorescence microscope and optical microscope, at 0.5, 1 or 2 hours after the start of incubation at 37 °C or 43 °C. Note that green signals indicate live cells and red signals indicate dead cells. **(b)** Ratios of dead U251 cells at 0.5, 1 or 2 hours after the start of incubation at 37 °C or 43 °C (n = 4, ns, not significant, ***p < 0.001). **(c)** Viability analysis without/with incubation at 43 °C for 1 hour in the presence of Fe(Salen) with saline in U251 cells. **(d)** Representative photon flux pictures of U251 cells in the presence of Fe(Salen) nanoparticles, without (*upper*)/with (*lower*) incubation at 43 °C for 1 hour. The viability of U251 cells was measured in terms of photon flux measured with IVIS. Note that cellular fluorescence was decreased in the presence of Fe(Salen) nanoparticles. **(e)** Photon flux (viability) of GB cells in the presence of Fe(Salen) nanoparticles, without/with incubation at 43 °C for 1 hour (n = 4, ****p < 0.0001).

We used an incubator to obtain the desired temperatures in this study (Supplemental Fig. 3), because AMF could not generate sufficient heat at the low level of Fe(Salen) concentration used for the *in vitro* assay (*data not shown*). Viability analysis with calcein-AM and propidium iodide staining showed that the number of dead U251 cells was unchanged at 37 °C, but was significantly increased in a time-dependent manner at 43 °C (Fig. 3a and b).

We previously reported that AMF-induced heating enhanced the cytotoxic effect of Fe(Salen)^27^. Therefore, we next evaluated the effect of incubation-induced hyperthermia on Fe(Salen) nanoparticle-induced cytotoxicity in U251 cells (Fig. 3c). When these cells were incubated at 43 °C in the presence of Fe(Salen) nanoparticles, growth inhibition was greater than with Fe(Salen) alone (Fig. 3d and e). Thus, the combination of Fe(Salen) and hyperthermia (43 °C) was more potent than either alone.

### Cytotoxic effects of Fe(Salen) and hyperthermia in normal human astrocytes (NHA)

**Figure 4:**
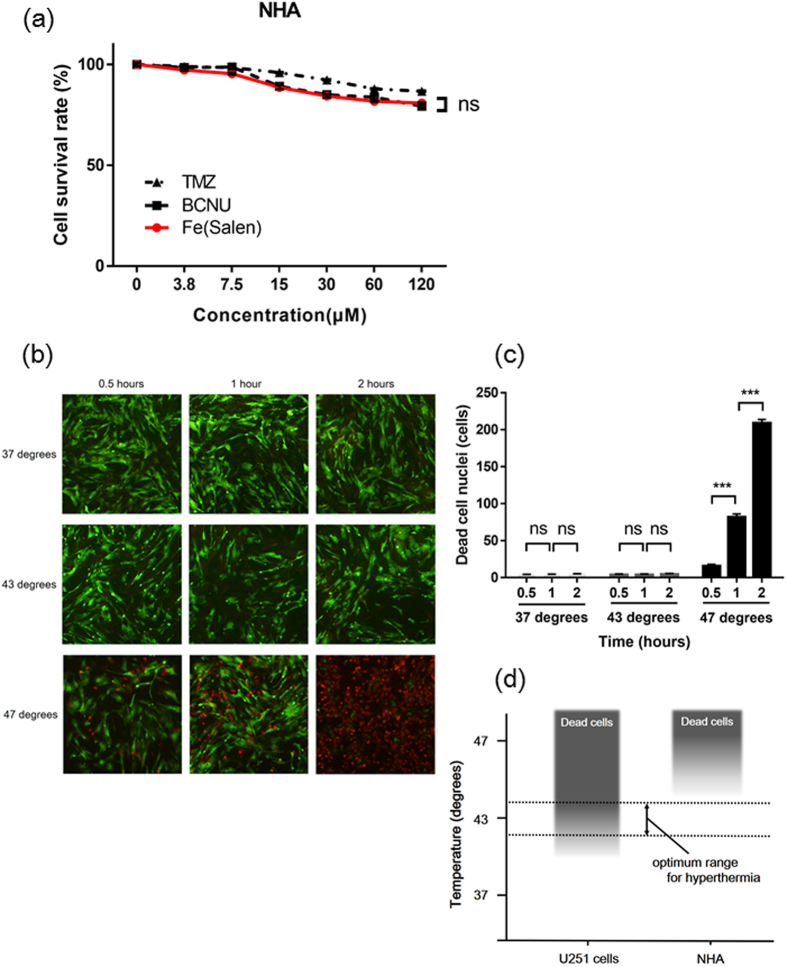
Cytotoxic effects of Fe(Salen) and hyperthermia in normal human astrocytes (NHA). (**a**) Effect of Fe(Salen) on proliferation of NHA in the presence of Fe(Salen), TMZ or BCNU (n = 4, ns, not significant). **(b)** Representative pictures of calcein-AM and propidium iodide staining in NHA at 0.5, 1 or 2 hours after the start of incubation at 37 °C, 43 °C or 47 °C. **(c)** Ratios of dead NHA at 0.5, 1 or 2 hours after the start of incubation at 37 °C, 43 °C or 47 °C (n = 4, ns, not significant, ***p < 0.001). **(d)** Optimum range of temperature for hyperthermia. Hyperthermia at 43 °C is harmful only to GB cells, not NHA.

In the treatment of brain tumor, it is very important to avoid injuring normal cells. We found that the cytotoxic effect of Fe(Salen) on NHA was similar to those of TMZ and BCNU (Fig. 4a). On the other hand, hyperthermia alone at 37 °C or 43 °C had little effect on the viability of NHA, although at 47 °C, the number of dead cells increased significantly in a time-dependent manner (Fig. 4b and c). These results show that hyperthermia at 43 °C is selectively cytotoxic to GB (Fig. 4d). Overall, the results in Figs 3 and 4 suggest that the combination of Fe(Salen) and hyperthermia at 43 °C might be selectively cytotoxic to GB.

### Systematic side effects and distributions of Fe(Salen) in the brain of rat

As our final goal is clinical application of Fe(Salen) in human brain, we first evaluated tissue distribution and systematic side effects following a single intracerebral injection of Fe(Salen). Our results indicate that the threshold dose for toxicity in rats (190–299 g) was between 0.12 mg/body and 0.60 mg/body in the case of single intracerebral injection (Supplemental Fig. 4a and b).

After single intracerebral injection of ^14^C-Fe(Salen) (0.12 mg/body) (Supplemental Fig. 5a), whole-body ARLGM revealed little loss of ^14^C-Fe(Salen) within 24 hours, with a substantial reduction by 168–504 hours (Supplemental Fig. 5b and c), in accordance with our previous report^26^. At a higher dose (0.32 mg/body), Fe(Salen) appeared to leak into cerebrospinal fluid, and was subsequently excreted to liver and kidney (Supplemental Fig. 5d).

We have previously reported that magnetic Fe(Salen) nanoparticles can be visualized by MR imaging^26^. Here, we demonstrated that MRI techniques enabled us to evaluate the distribution of Fe(Salen) in rat brain after a single intracerebral injection (Supplemental Fig. 6a). Fe(Salen) remained detectable for more than 3 days (Supplemental Fig. 6b).

### Fe(Salen) suppresses tumor growth and AMF enhances the anti-tumor effect *in vivo*

Based on the above findings, we next examined whether the combination of Fe(Salen) and 43 °C hyperthermia induced by AMF would be effective in a mouse model grafted with GB cells onto the left leg. We have previously reported that AMF-induced heating of Fe(Salen) nanoparticles results in enhanced cytotoxicity in a tongue cancer xenograft model prepared with human squamous cancer cells^27^. In this study, we selected a leg graft GB model to evaluate the effect of simultaneous hyperthermia-chemotherapy using Fe(Salen) nanoparticles, because the small brain volume of mice limits the acceptable volume of Fe(Salen) injection.

**Figure 5:**
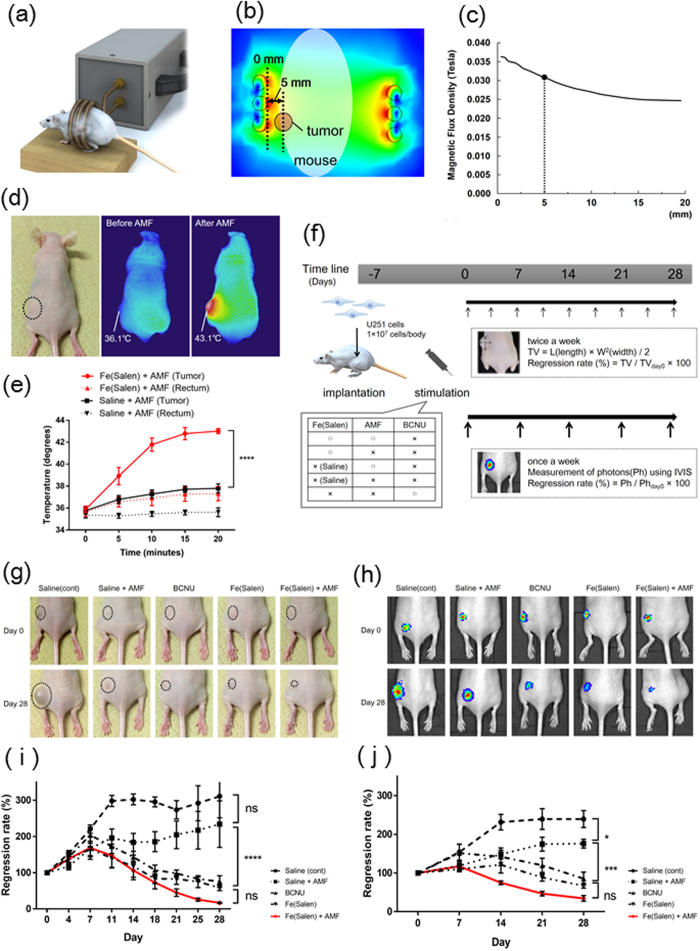
Local injection of Fe(Salen) nanoparticles generates heat upon exposure to AMF, and this heat enhances the anti-tumor effect in the GB mouse model. (**a**) Illustration of a mouse in the coil of the AMF generator. (**b**) Magnetic flux density distribution from the simulation (AMF: 280 kHz and 335.4 Arms). Colors indicate the magnetic flux density. The distance between the tumor and the edge of coil, and the estimated magnetic flux density is shown. (**c**) Relationship between the magnetic flux density and the distance of the tumor from the edges of the coil during AMF exposure (280 kHz and 335.4 Arms). (**d**) Representative thermography image at the tumor site (*left*) after injection of Fe(Salen) nanoparticle suspension before (*middle*)/after AMF exposure (*right*). **(e)** Temperature change at the rectum and the injection site of Fe(Salen) suspension with saline or saline upon AMF exposure. Either Fe(Salen) nanoparticle suspension or saline was injected into the tumor in mice, followed by exposure to AMF. Note that the local temperature was time-dependently increased to a greater degree with Fe(Salen) (n = 6, ****p < 0.0001). (**f**) Treatment schedule for GB mouse experiment. (**g**) Representative pictures of mouse leg tumor in each treatment group. (**h)** IVIS images of mouse leg tumor in each treatment group at Day 0 *(upper*) and Day 28 (*lower*). (**i**) Regression rate based on manual measurement of tumor volume changes in mouse leg. The red line indicates the ratio in the Fe(Salen) with AMF exposure group (n = 6, ns, not significant, ****p < 0.0001). (**j**) Regression rate based on photon flux measurement of tumor volume changes in mouse leg. The red line indicates the ratio in the Fe(Salen) with AMF exposure group (n = 6, ns, not significant, *p < 0.05, ***p < 0.001).

To confirm that AMF can increase the temperature to 43 °C, Fe(Salen) nanoparticles were injected subcutaneously into the area around the GB tumor site on the left leg. The mouse was placed in the coil and exposed to AMF (280 kHz and 335.4 Arms) (Fig. 5a). The distance between the tumor and the edge of the coil was 5 mm (Fig. 5b). The estimated magnetic flux density was about 0.031 Tesla at the tumor site and was fairly uniform (Fig. 5c). The temperature at the subcutaneous injection site was measured by thermography, which showed an increase of temperature to a maximum of 43.1 °C (Fig. 5d), and with a thermometer (Fig. 5e). The tip of the microfiber of the thermometer was inserted into the center of the tumors and then the change of temperature was measured. The local temperature was time-dependently increased to a greater degree with Fe(Salen) (Fig. 5e). The rectal temperature of Fe(Salen) group was slightly increased to average 37.7 °C (Fig. 5e), which might be influenced by the blood circulation warmed up by the drug heat generation. Thus, AMF could generate a sufficient heating effect for hyperthermia treatment. In contrast, the temperature remained below 39 °C in the AMF-exposed saline control.

At 7 days after implantation of U251 cells transfected with luciferase-encoding vector, mice were segregated into groups as shown in Fig. 5f, and tumor growth was monitored for 4 weeks by IVIS. Tumor size was evaluated both manually (Fig. 5g) and by measurement of photon flux with IVIS (Fig. 5h). Tumor size in the control group increased about 300% after 4 weeks. In the saline with AMF group, tumor size was slightly, but not significantly, smaller than in the control group. Tumor sizes in both the Fe(Salen) and BCNU subcutaneous injection groups were significantly reduced by about 50% compared to the control group at 4 weeks. The tumor size was most markedly decreased, by 80–90%, in the Fe(Salen) plus AMF group. However, the difference between the Fe(Salen) plus AMF group and the Fe(Salen) alone group was not statistically significant (Fig. 5i). Similar results were obtained by photon flux measurement (Fig. 5j). Histological examination by HE (Supplemental Fig. 7a) and Ki-67 staining (Supplemental Fig. 7b) confirmed that the tumors showed the characteristics of GB.

## Discussion

Our results indicate that the chemotherapy with Fe(Salen) nanoparticles and AMF-induced hyperthermia exhibited an effective anti-tumor effect in a leg tumor model of GB. This strategy produced a greater decrease of tumor size than did either BCNU alone or Fe(Salen) alone, though the difference was not statistically significant, probably because of the highly individual difference. Conventional inorganic particles including iron-oxide nanoparticles (Fe_3_O_4_) for clinical use lack intrinsic anti-tumor effect^25^. In contrast, Fe(Salen) has not only strong anti-tumor effect but also hyperthermia-inducing effect in an AMF irradiation, therefore, the use of Fe(Salen) nanoparticles could be done for anti-tumor and hyperthermia therapies at the cancer site. This is a major advantage compared to the conventional treatment.

We have set out to develop a reproducible protocol for synthesis of Fe(Salen) nanoparticles with consistent purity and magnetic properties in line with Good Manufacturing Practice (GMP), as recommended by the International Conference on Harmonisation of Technical Requirements for Registration of Pharmaceuticals for Human Use (ICH) (Hoshino *et al*. *unpublished data*).

Other issues also remain before clinical application will be possible. First, the origin of the magnetic properties of Fe(Salen) needs further investigation. Although Fe(Salen) possesses a crystal structure that accords with the classical Goodenough-Kanamori-Anderson rule regarding magnetic interactions in the Fe complex^26^,^35^, it is not clear whether this is necessary or sufficient. Further collaborative investigation with chemist and physicists may be necessary in order to develop suitable drug compounds to deliver hyperthermia. Moreover, it may be necessary to optimize the magnetic properties for clinical application. In our preliminary study, the values of “Specific Loss Power” of Fe(Salen) are 2.7 W/g with 100 mM (AMF: 300 kHz and 300.3 Arms) and 3.8 W/g with 200 mM (AMF: 300 kHz and 300.0 Arms). One of the reason of such lower SLP is Fe(Salen) has smaller value of magnetic saturation compared to those of traditional magnetite or other ferrite-based nanoparticles. In addition to the value of SLP, the stronger magnetic properties compared to Fe(Salen) itself need further investigation and developing e.g. micelles coating with Fe(Salen).

Secondly, the therapeutic protocol for hyperthermia and the AMF generator need to be optimized for human patients. Our tentative calculations based on available data indicate that we need a more powerful AMF generator, which can produce a higher electric current, so as to generate enough heat for efficient hyperthermia treatment. We are currently working for improving the AMF generator and coil design.

Thirdly, we demonstrated AMF-induced Fe(Salen)-mediated heat generation in the leg tumor mouse model, but not in the brain model because of machine limitation and small capacity of cranial space of mice. According to the toxicity test, 10 mM is the maximum concentration of Fe(Salen) which can be injected in mouse brain without major side effects (Supplemental Figs 4 and 5). Moreover, the permissible volume of local injection of Fe(Salen) in mouse brain, i.e., 10 μl, is much smaller than that of in the leg tumor model. This injection volume in mouse brain was thus too small to generate heat for effective hyperthermia treatment. The maximum temperature was 36.8 °C in mice brain injected 10 μl Fe(Salen) when exposed to an AMF. Therefore, the regression rate of brain tumor was not significantly different between Fe(Salen) with AMF and Fe(Salen) without AMF at 28 days after stimulation. This was due to insufficient increase in the temperature for hyperthermia (Supplemental Fig. 8a).

We have thus examined the volume necessity for the local injection of Fe(Salen) that generates heat for effective hyperthermia treatment in mouse brain (Supplemental Fig. 8b). The result indicated that more than 500 μl Fe(Salen) (10 mM) should be needed for enough heat elevation up to 43.0 °C, which could not be achieved in mouse brain. Because the sufficient volume for hyperthermia was permitted in the leg, we used a leg tumor model to evaluate the dual strategy *in vivo* in the current study. In the future, we should use a large animal model, which is permitted higher volume injection into the brain, in order to examine the dual strategy in the brain tumor model. However, it is difficult to demonstrate the dual therapy using mouse brain model in the current study.

Considering the above, we still need to perform further study and optimize the magnetic properties of Fe(Salen), the specifications of more powerful AMF generator, and the examination using an animal brain tumor model, for human application. However, in this study, we have indicated, at least, the new direction of GB treatment *in vitro* and *in vivo*, hyperthermia and chemotherapy with single-drug Fe(Salen) nanoparticles.

## Additional Information

**How to cite this article:** Ohtake, M. *et al*. Hyperthermia and chemotherapy using Fe(Salen) nanoparticles might impact glioblastoma treatment. *Sci. Rep.* **7**, 42783; doi: 10.1038/srep42783 (2017).

**Publisher's note:** Springer Nature remains neutral with regard to jurisdictional claims in published maps and institutional affiliations.

## Supplementary Material

Supplementary Materials
