# Supplementary material for: Hyperthermia and chemotherapy using Fe(Salen) nanoparticles might impact glioblastoma treatment
Source: Sci Rep. 2017 Feb 20;7:42783. doi: 10.1038/srep42783 (PMC5316938; doi:10.1038/srep42783)
Supplement: Supplementary Materials [file srep42783-s1.docx]

Supplementary Materials for

**Hyperthermia and chemotherapy using Fe(Salen) nanoparticles might impact glioblastoma treatment**

Makoto Ohtake, Masanari Umemura, Itaru Sato, Taisuke Akimoto, Kayoko Oda, Akane Nagasako, Jeong-Hwan Kim, Takayuki Fujita, Utako Yokoyama, Tomohiro Nakayama, Yujiro Hoshino, Mai Ishiba, Susumu Tokura, Masakazu Hara, Tomoya Muramoto, Sotoshi Yamada, Takatsugu Masuda, Ichio Aoki, Yasushi Takemura, Hidetoshi Murata, Haruki Eguchi, Nobutaka Kawahara and Yoshihiro Ishikawa

**This PDF file includes:**

Figs. S1 to S8


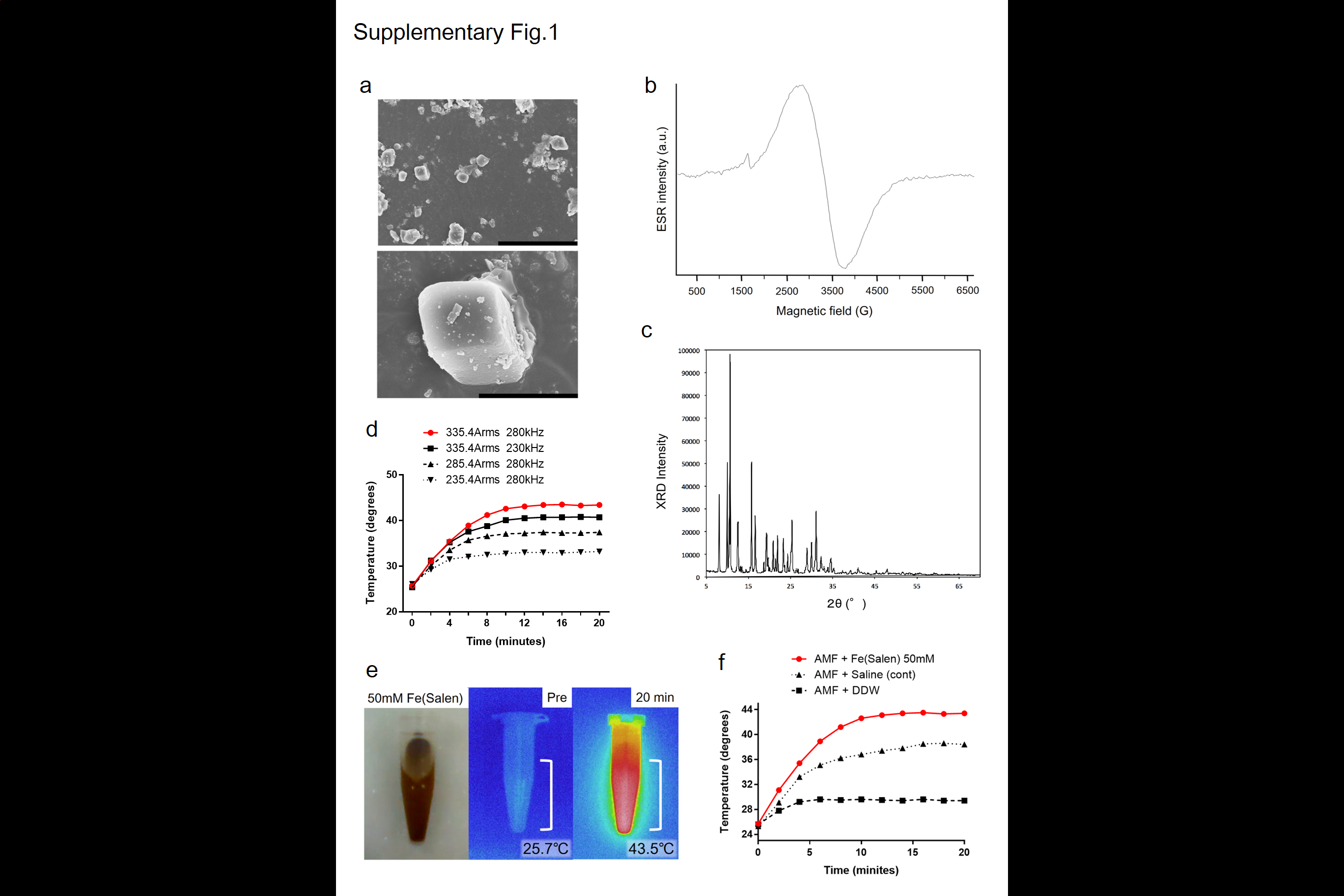


**Supplemental Figure 1. Particle size and magnetism of Fe(Salen) solution (50 mM)**

(**a**) Scanning electron microscope images of extensively sonicated (6 hours) Fe(Salen) nanoparticles. Scale bar = 30 µm (*upper*), 5 µm (*lower*).

(**b**) Electron paramagnetic resonance (EPR) spectra for Fe(Salen) solution 50 mM was measured with an X-band ESR spectrometer (Bruker EMX, Bruker BioSpin, Germany). Microwave power and modulation amplitude were 1 mW and 1 G, respectively.

(**c**) The powder XRD characterization of Fe(Salen) sample.

(**d**) Temperature measurement of 50 mM Fe(Salen) solution with saline at different frequencies and currents.

(**e**) Representative thermographic images to show heat generation by Fe(Salen) solution 50 mM in a tube upon exposure to an alternating magnetic field at a frequency of 280 kHz and a current of 335.4 Arms for 20 minutes (Pre, before exposure; 20 minutes, after exposure for 20 minutes).

(**f**) A time-dependent temperature measurement of 50 mM Fe(Salen) solution with saline compared with saline as the suspension medium of Fe(Salen), and deionized distilled water (DDW).


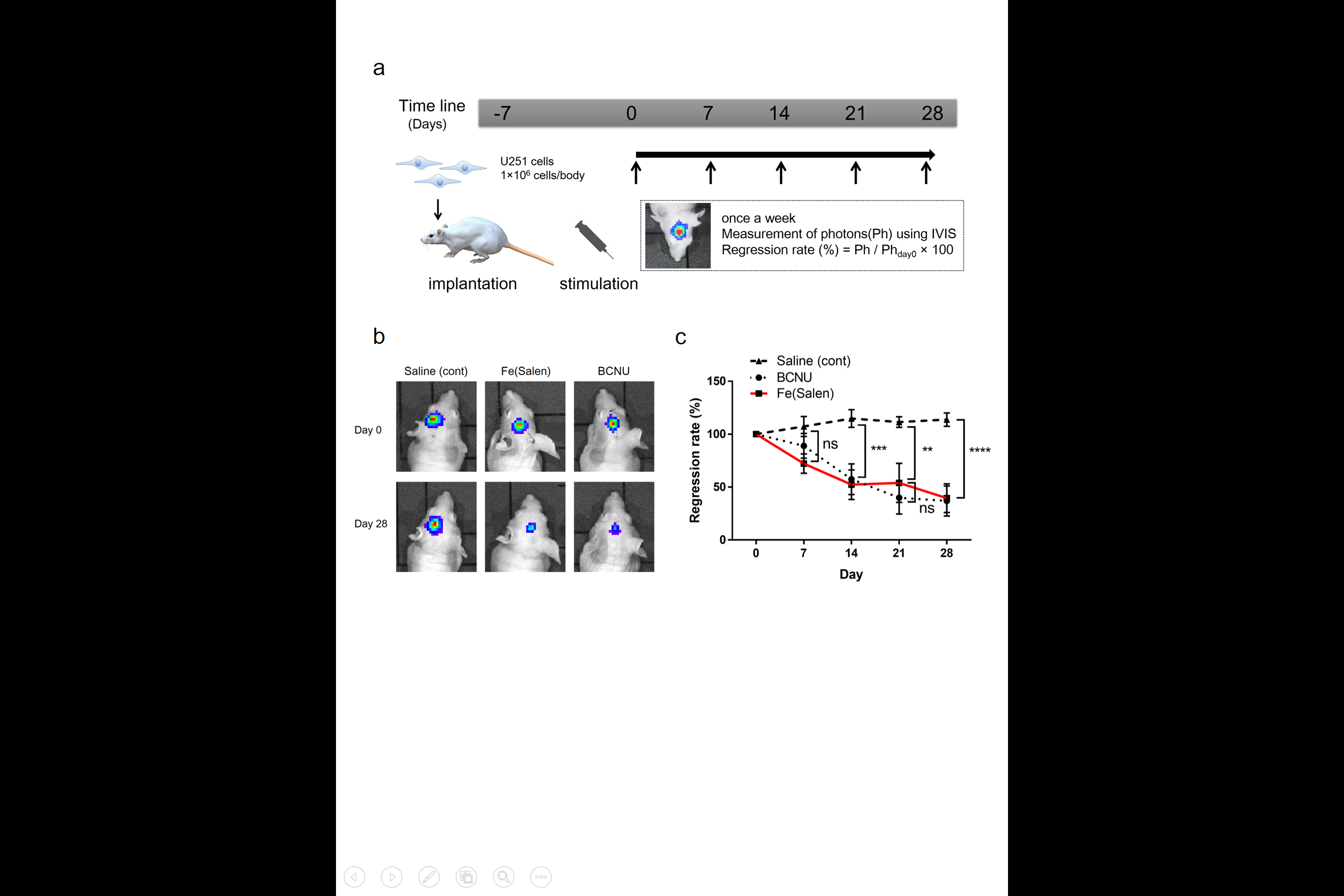


**Supplemental Figure 2. Fe(Salen) nanoparticles suppress tumor growth in brain of mice**

(**a**) Treatment schedule for mouse brain model of GB.

(**b**) Representative IVIS imaging of mouse brain at day 0 (upper) and day 28 (lower) in saline (control) (*left*), Fe(Salen) (*middle*) and BCNU (*right*) intracerebral injection groups.

(**c**) Comparison of tumor volume ratios in the control (saline), Fe(Salen) and BCNU injection groups at 28 days. The red line indicates the ratio in the Fe(Salen) group (n=4, ns, not significant, ***p*<0.01, ****p*<0.001, *****p*<0.0001).


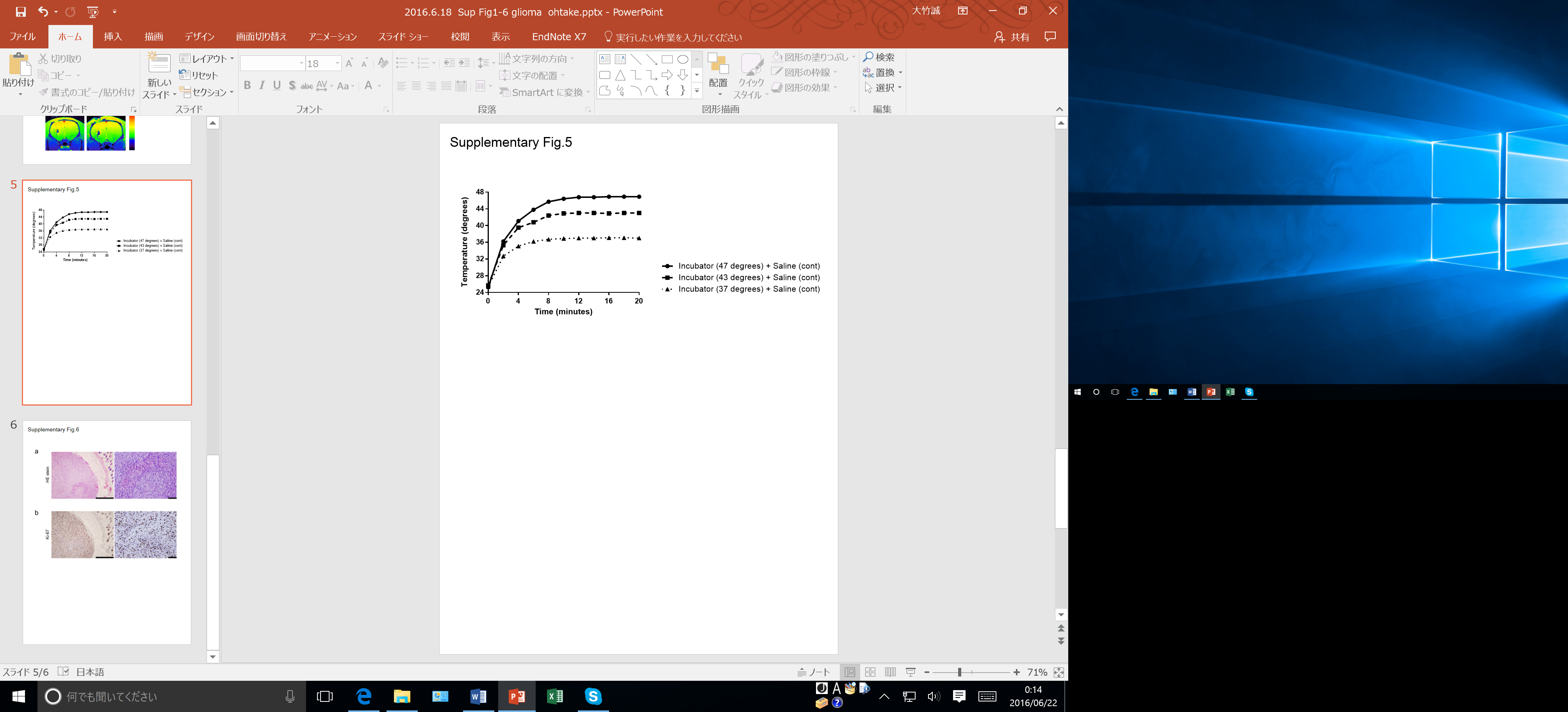


**Supplemental Figure 3. Temperature changes of saline at different conditions**

Changes of temperature of saline in a culture dish initially at room temperature in an incubator set at 37 °C, 43 °C or 47 °C. The target temperature was reached in about 10 minutes.

**
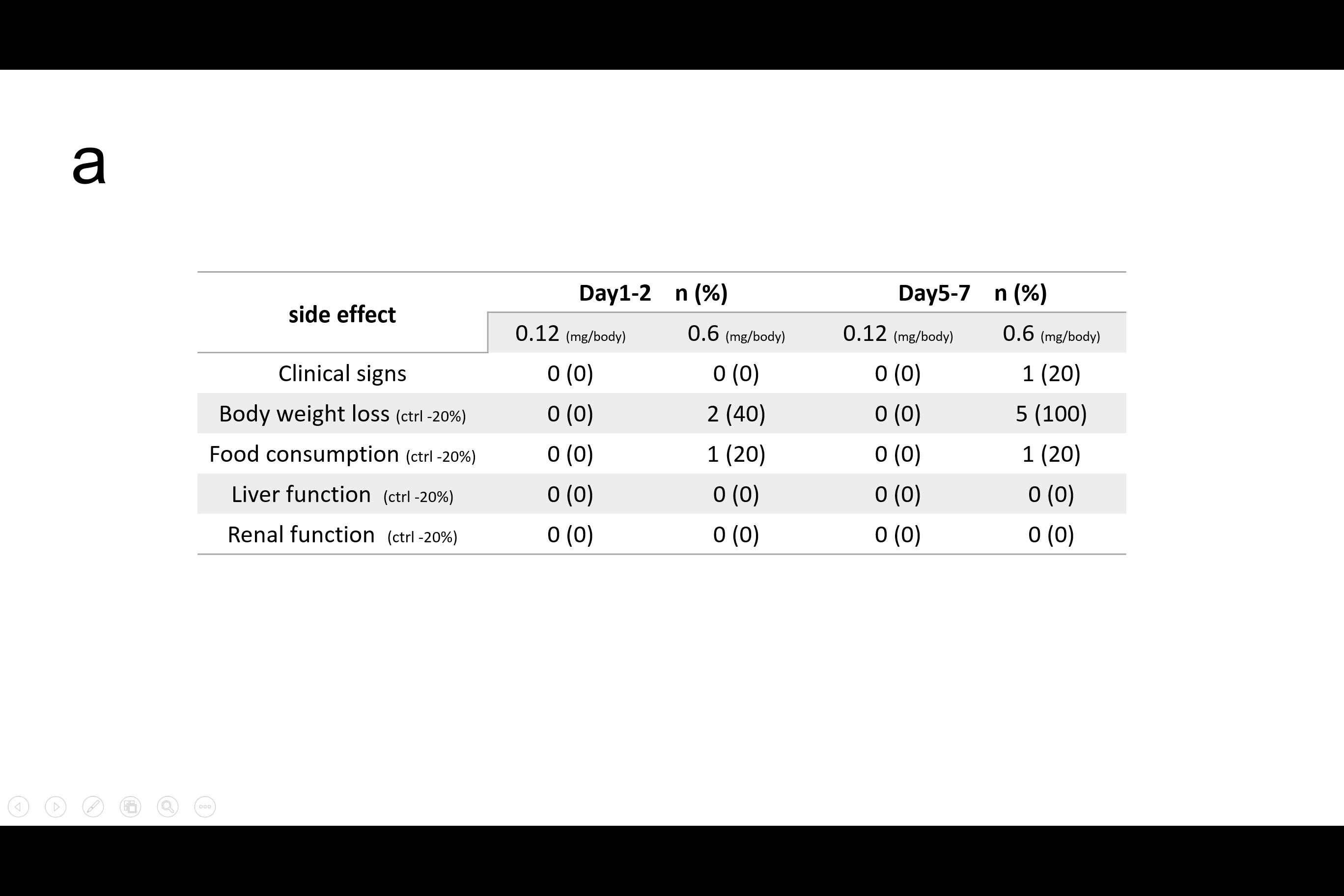
**

a

b


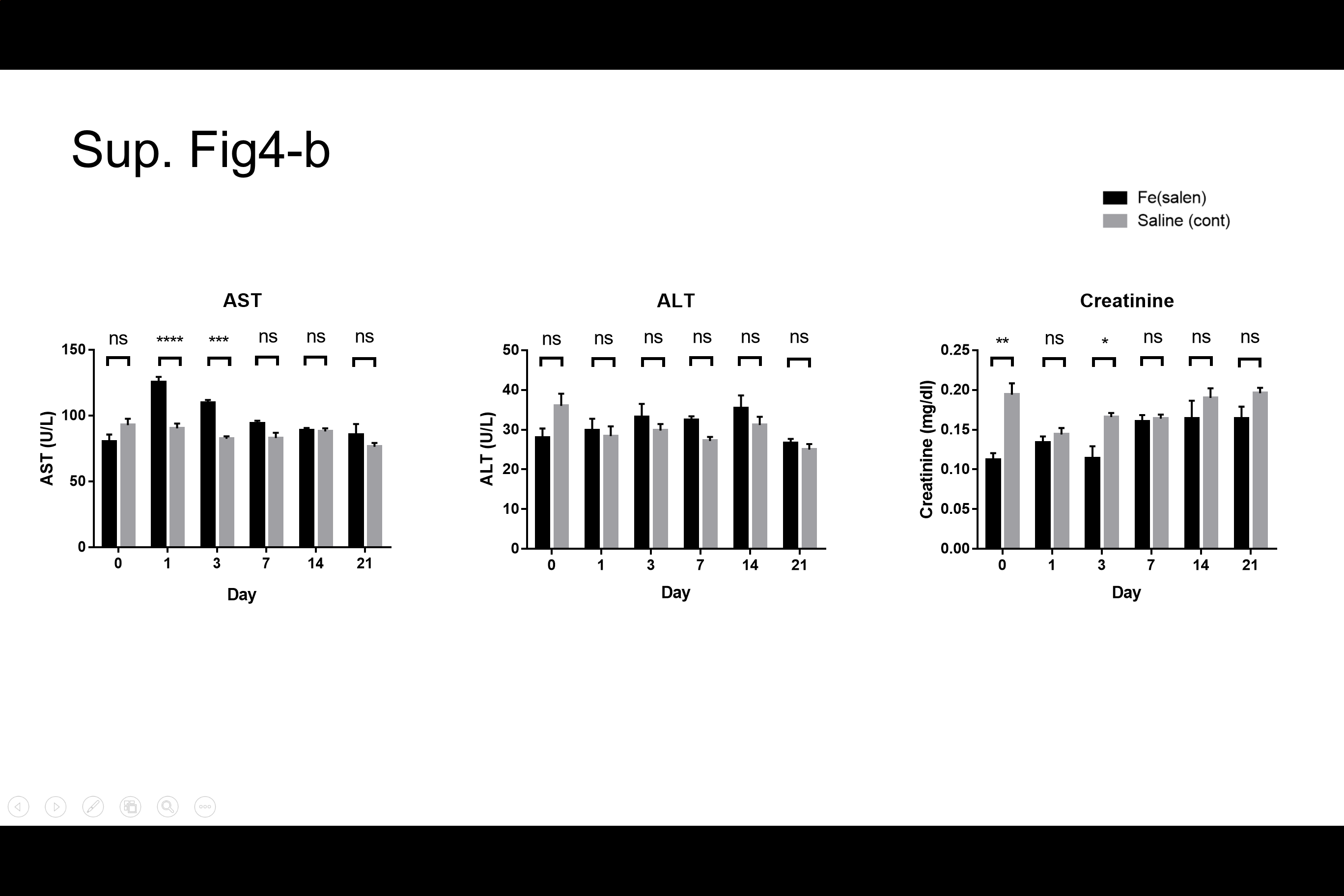


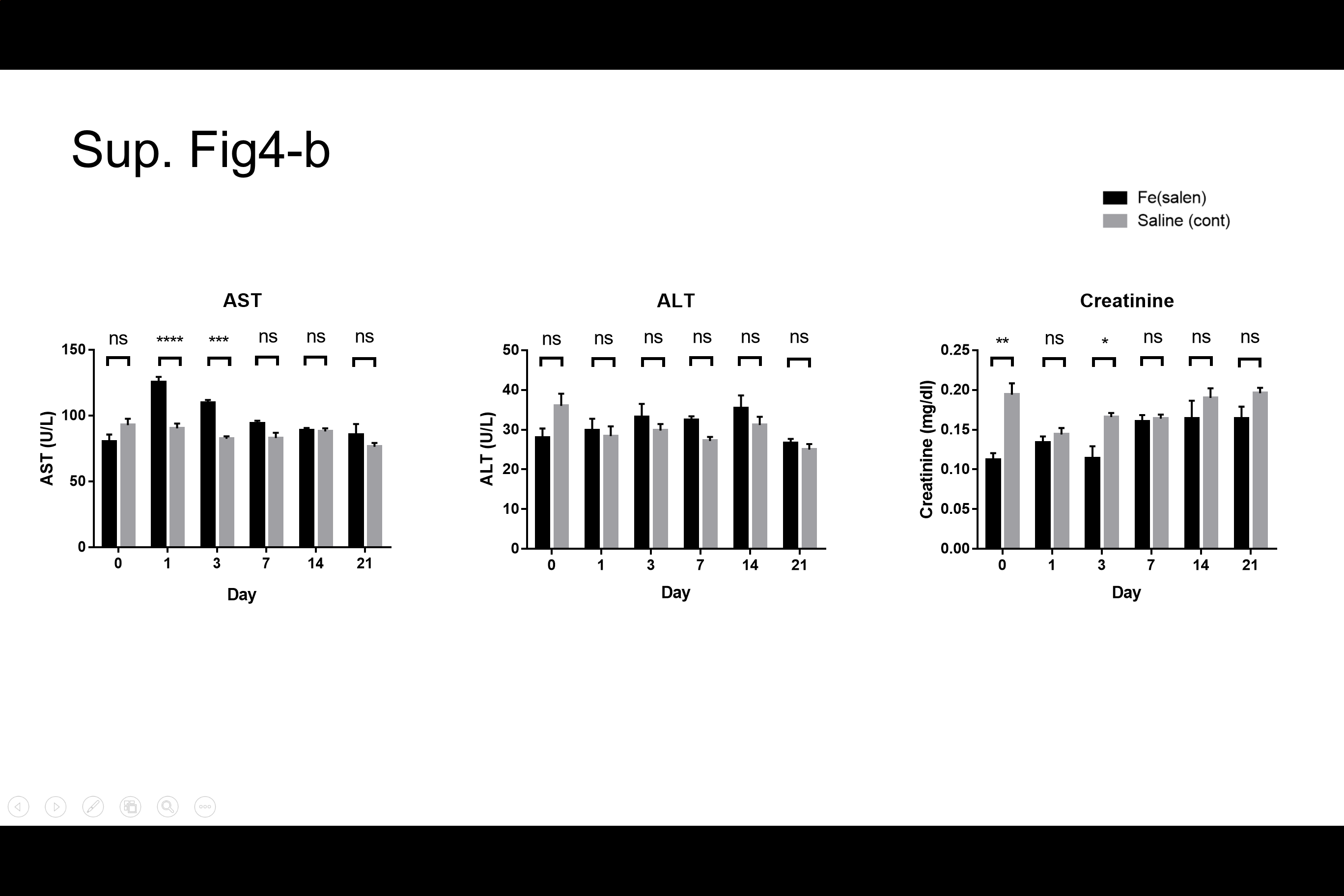


**
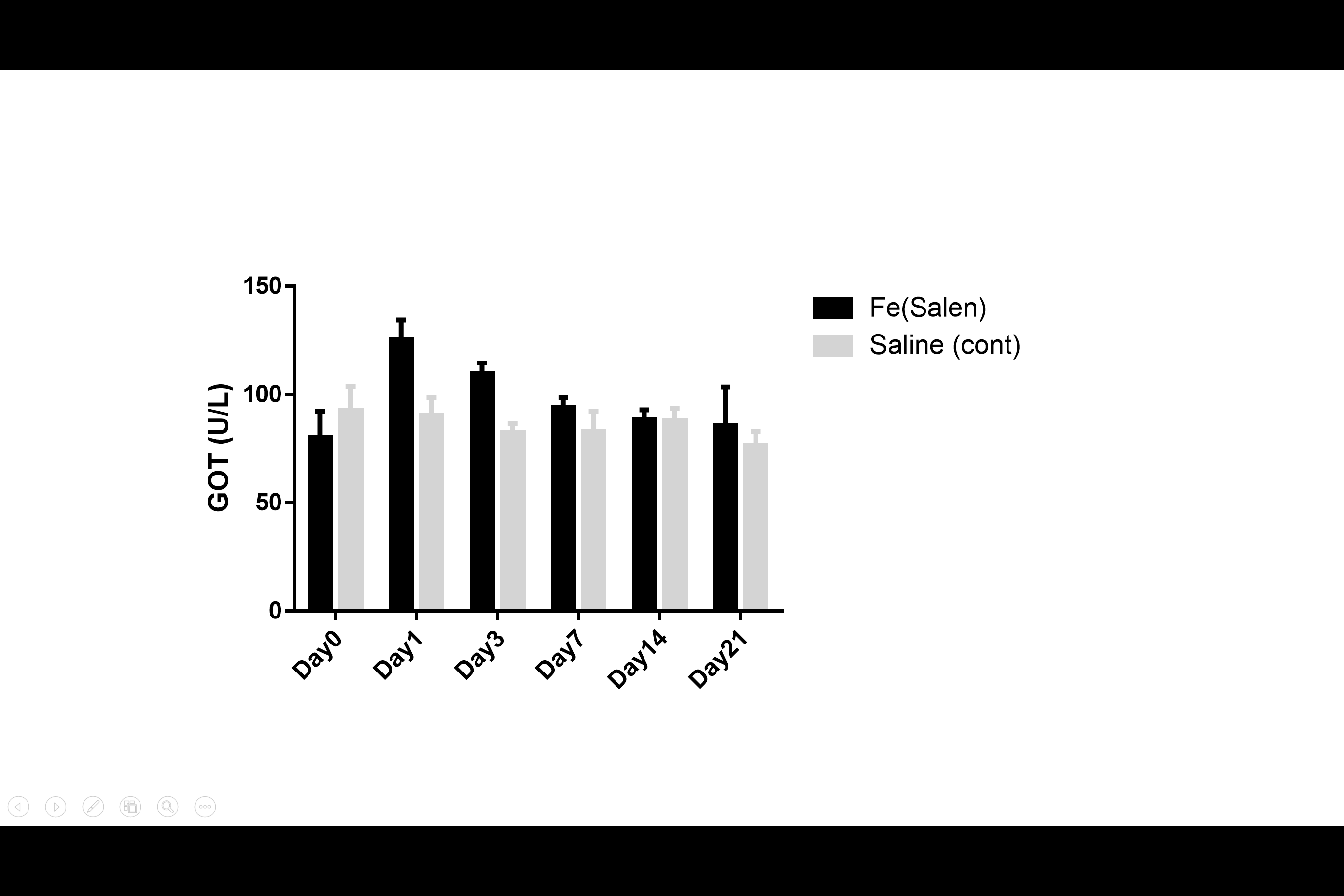
**

**Supplemental Figure 4. Examination of systematic side-effects of Fe(Salen) after injection into rat brain**

(**a**) Toxicity was examined by evaluating body weight loss, food consumption, and liver and renal functions at 1-2 days and 5-7 days after Fe(Salen) nanoparticle injection (0.12, 0.60 mg/body) into the brain of rats. A difference of at least 20% from the control was taken as a potential side effect. At 5-7 days after injection, all rats in 0.60 mg/body group showed more than 20 % decrease of body weight versus the controls. Clinical signs, including activity, were also evaluated; one rat in the 0.60 mg/body group had a foreleg injury, but this appeared unrelated to the treatment.

(**b**) Changes in parameters of liver function (AST, ALT) and renal function (creatinine) were examined just before Fe(Salen) injection (0.60 mg/body) into the brain of rats and 1 day, 3 days, 7 days, 14 days and 21 days after injection. Serum AST was significantly increased at 1-3 days after injection of Fe(Salen), but normalized thereafter. Creatinine was significantly greater in the treated group than in the control before and at 3 days after injection, but was still within the normal range (n=5, ns, not significant, **p*<0.05, ***p*<0.01, ****p*<0.001, *****p*<0.0001).


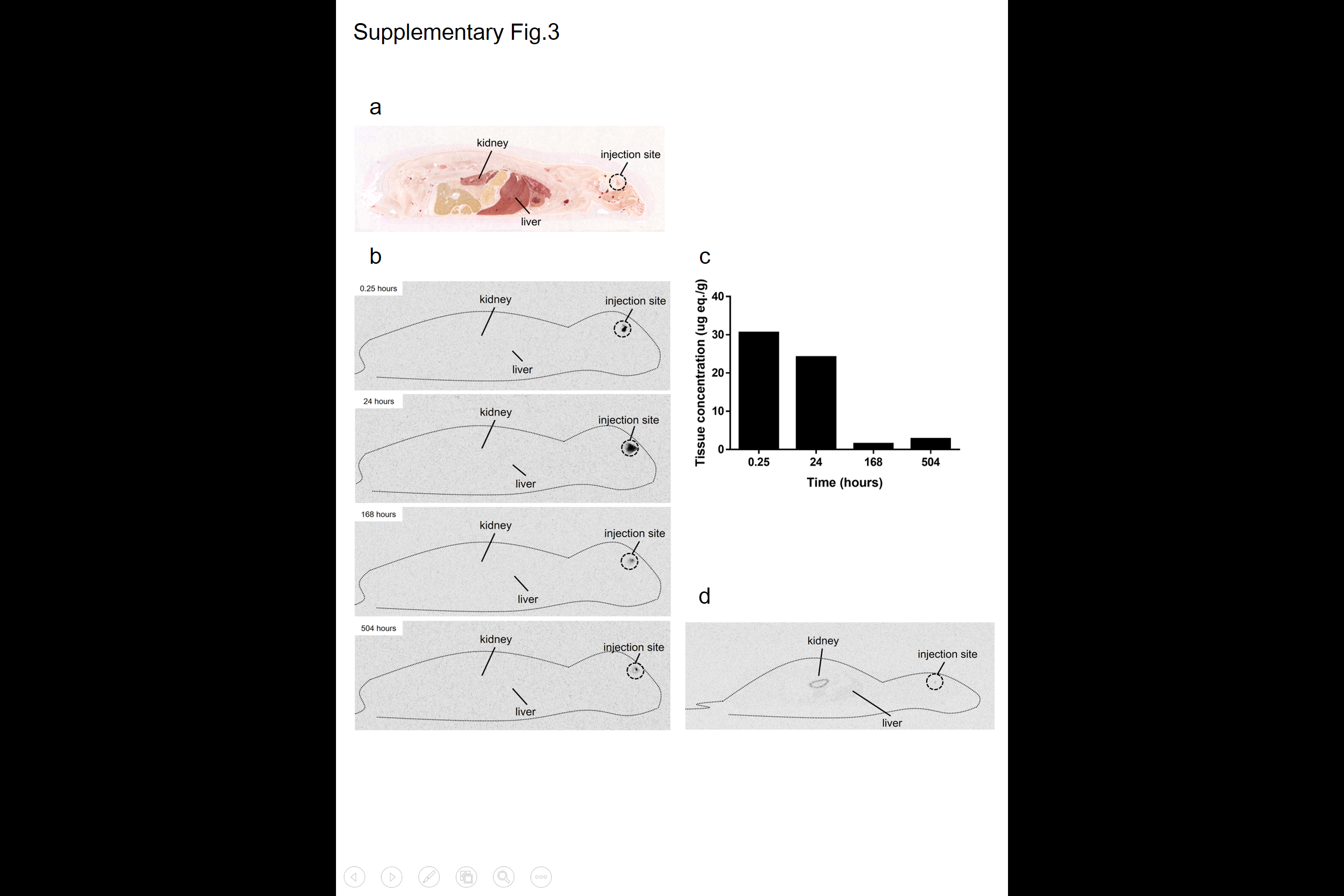


**Supplemental Figure 5. Distribution of ^14^C-Fe(Salen) in rats after injection into the brain**

(**a**) Frozen whole-body section of rat.

(**b**) Results of whole-body autoradioluminography (ARLGM) at 0.25 hours, 24 hours, 168 hours, and 504 hours after a single intracerebral administration of ^14^C-Fe(Salen) at 0.12 mg/body in a male rat. Representative images of tissue distribution from 0.25 hours to 504 hours are shown.

(**c**) Tissue concentration in brain from 0.25 hours to 504 hours after single intracerebral injection of ^14^C-Fe(Salen) (0.12 mg/body) (n=1).

(**d**) Whole-body ARLGM image obtained at 3 hours after a single intracerebral administration of ^14^C-Fe(Salen) at the higher dose of 0.32 mg/body, at which leakage of ^14^C-Fe(Salen) into cerebrospinal fluid occurred. ^14^C-Fe(Salen) was distributed mainly to the kidney and slightly to the liver.


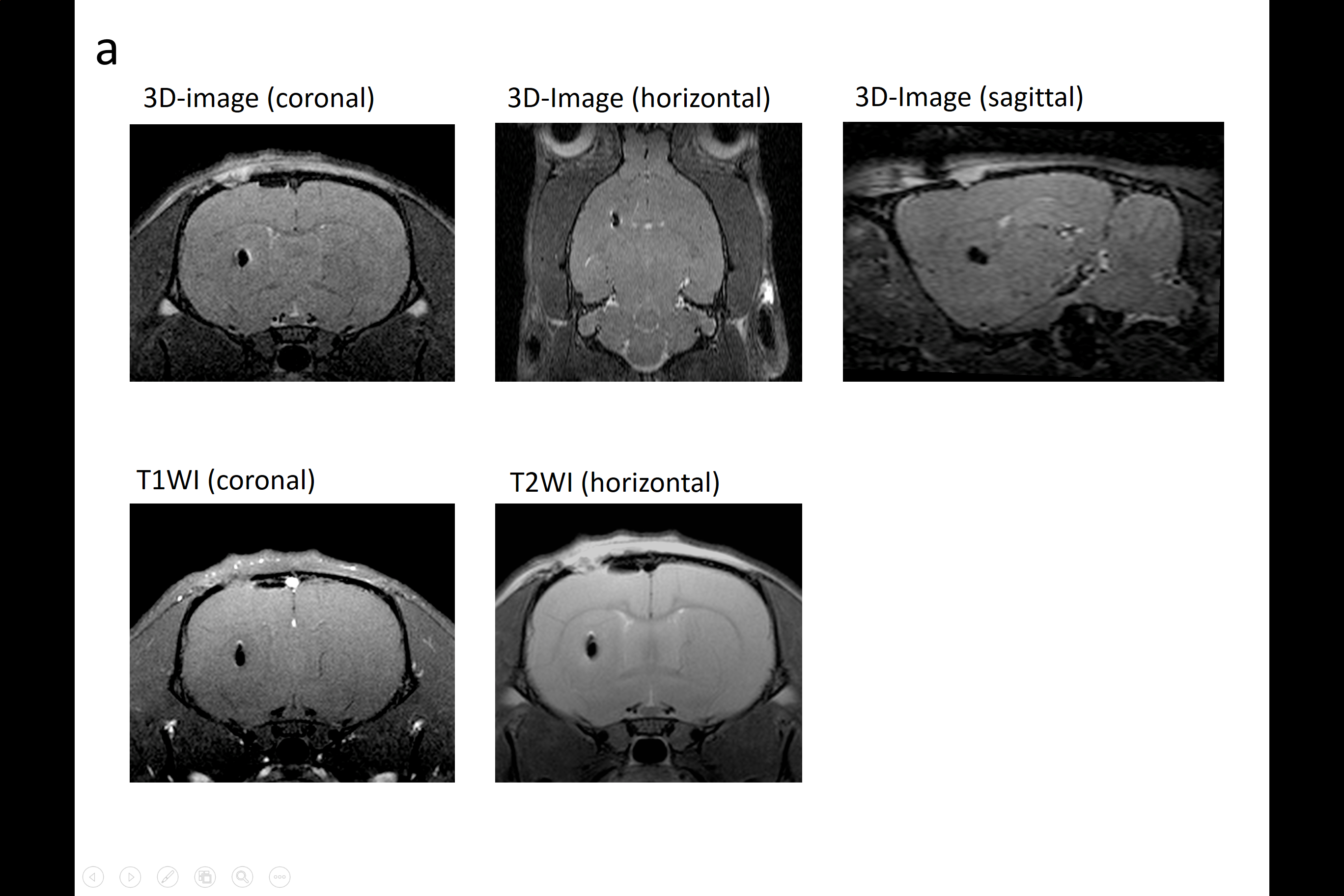


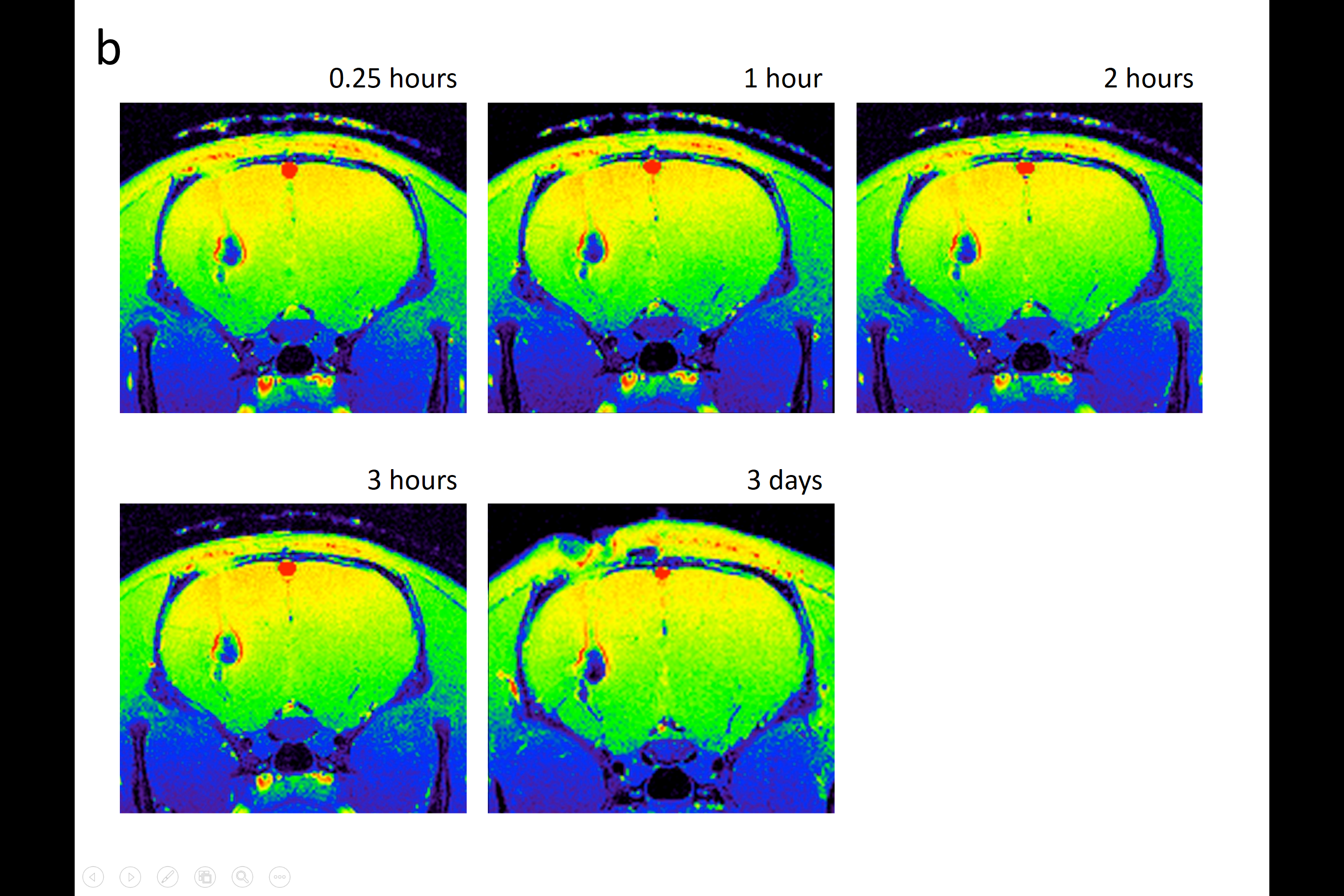


**Supplemental Figure 6. Magnetic resonance imaging of Fe(Salen) in rat brain**

(**a**) T1-weighted and T2-weighted images clearly visualized the localization of Fe(Salen) in the brain.

(**b**) T1-weighted MRI scans at 0.25 hours, 1 hour, 2 hours, 3 hours, and 3 days after injection of Fe(Salen) showed almost no change, indicating that Fe(Salen) is retained at the injection site for at least 3 days.

**
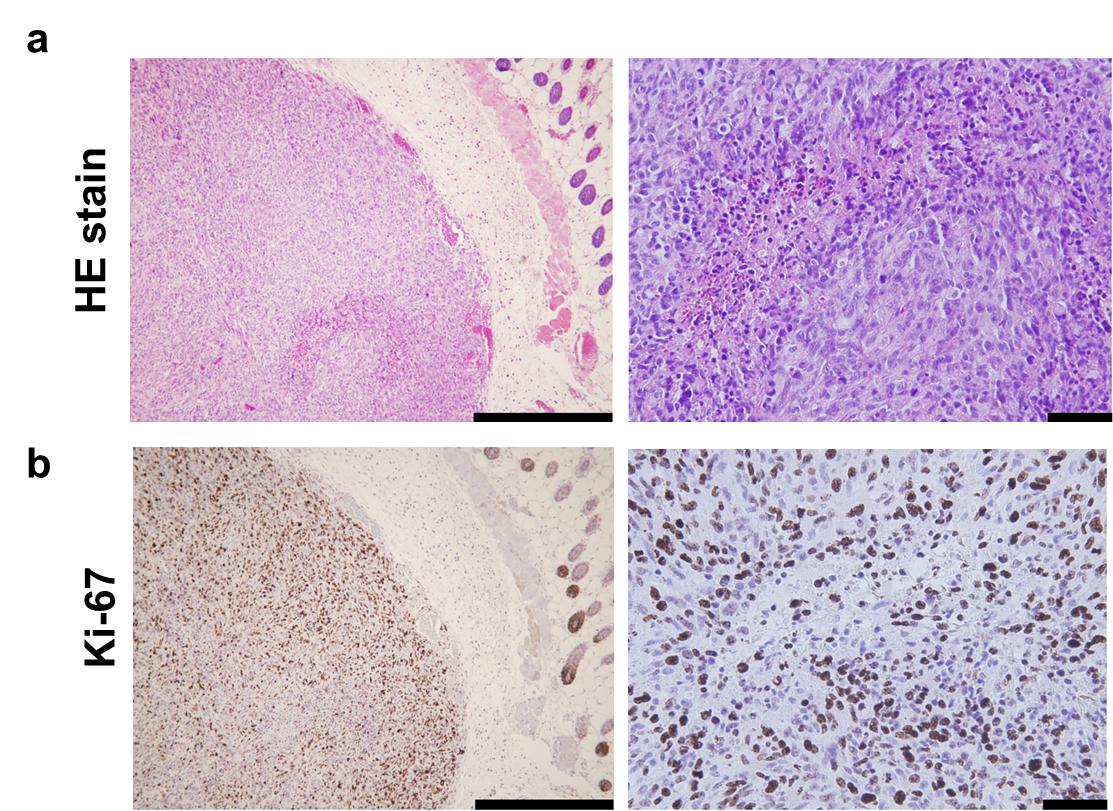
**

**Supplemental Figure 7. Histology of hypodermal GB model**

(**a**) Representative histopathological images (HE staining) of hypodermal GB model (day 28). Atypia, microvascular proliferation and necrosis, characteristic of GB, are seen. Scale bar = 500 µm (*left*), 50 µm (*right*).

(**b**) Tissue sections were immunostained with anti-Ki-67 antibody. Scale bar = 500 µm (*left*), 50 µm (*right*). High concentrations of Ki-67 positive cells are seen.


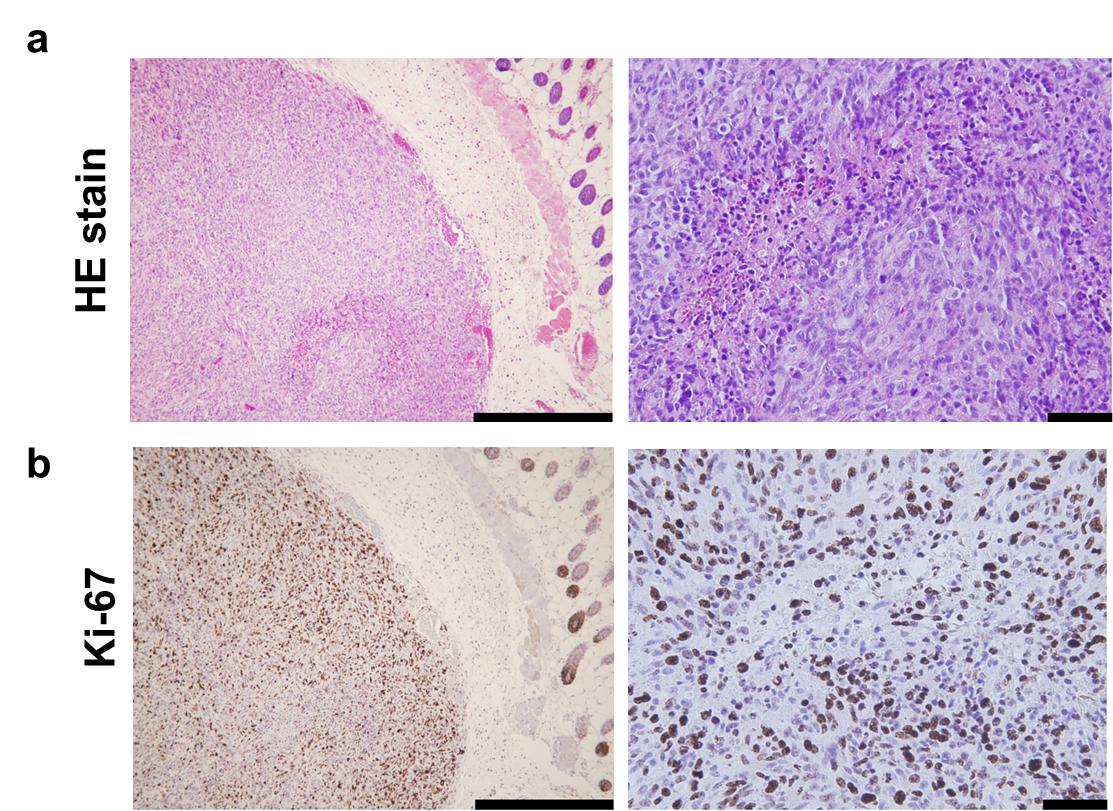

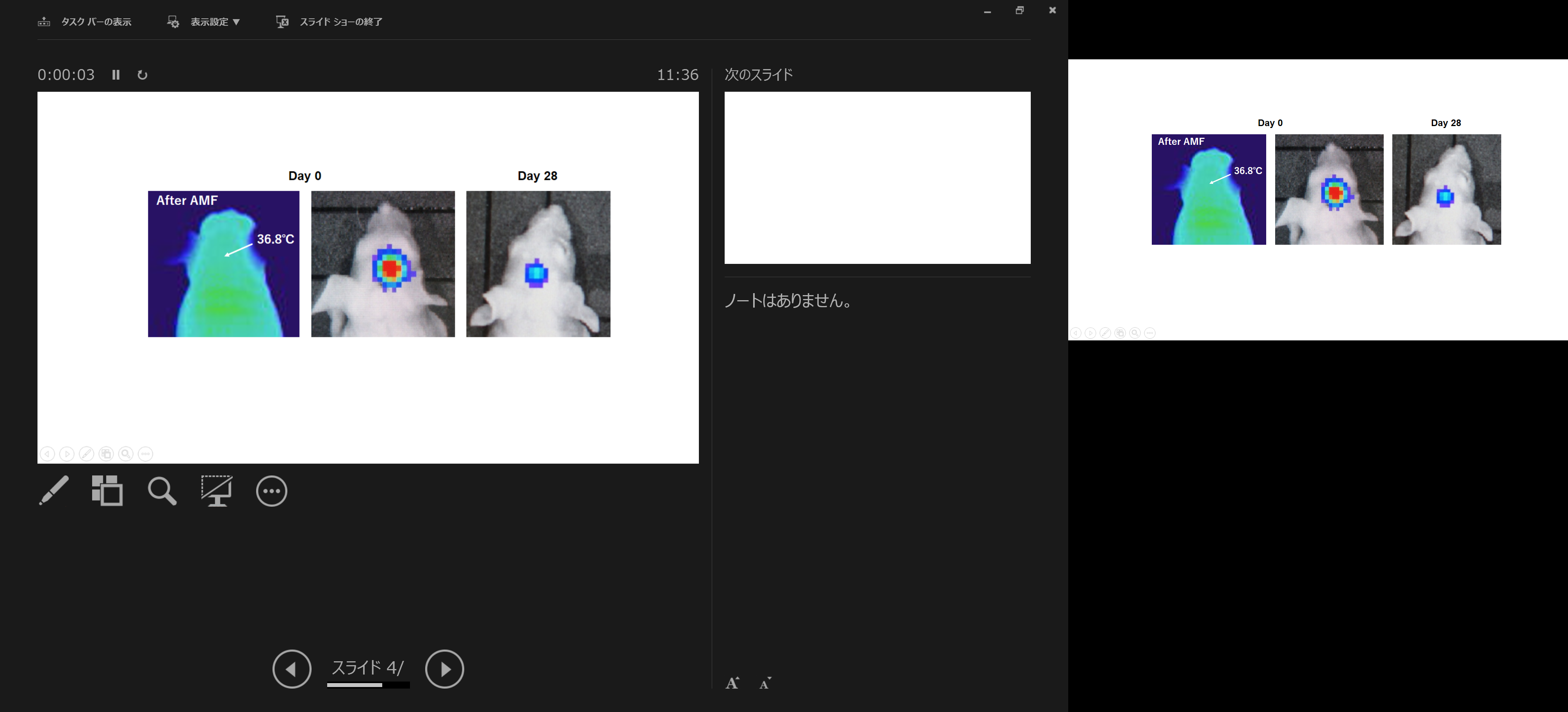


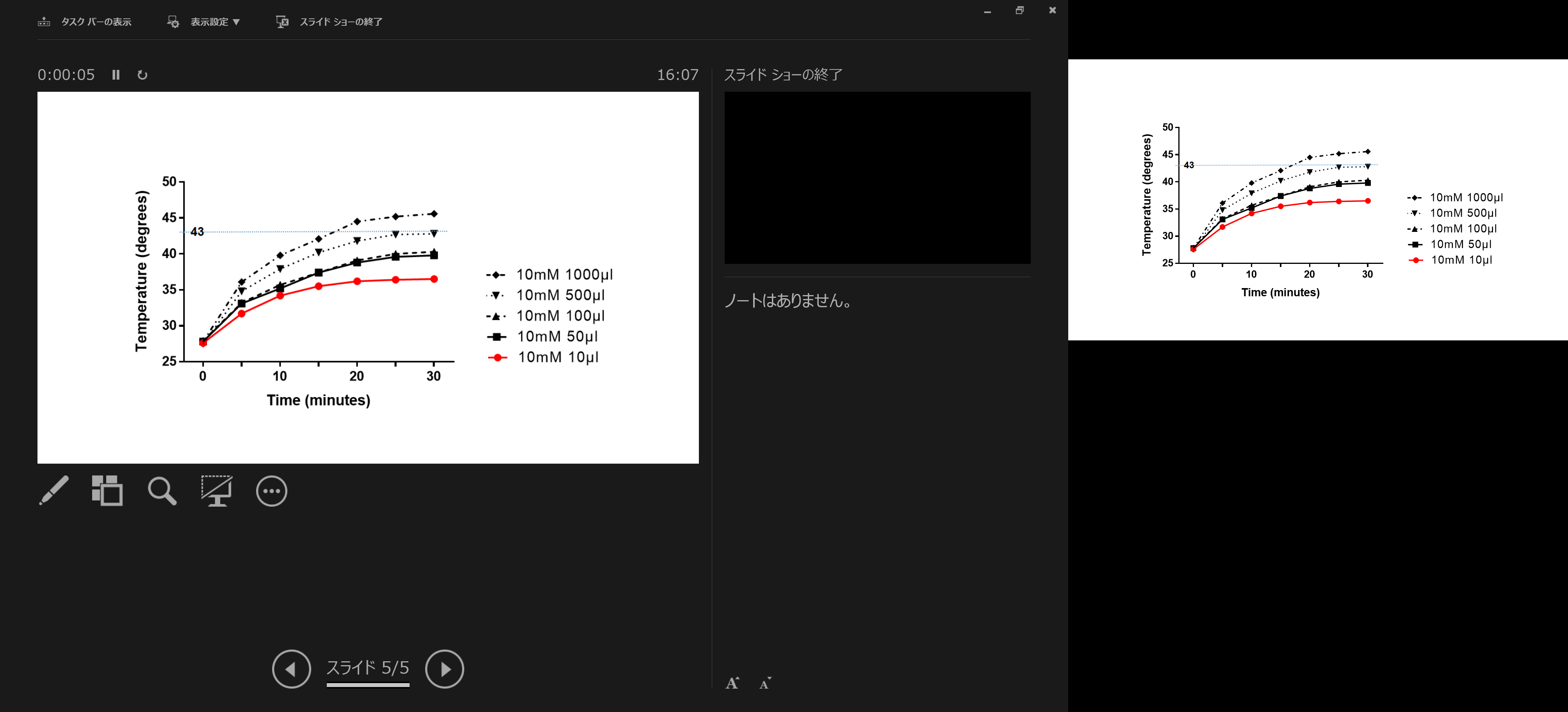


**Supplemental Figure 8. Fe(Salen) (10 mM/10 µl) cannot generate enough heat for hyperthermia in mice brain model**

(**a**) Representative thermographic imaging (*left*) and IVIS imaging of mouse brain at day 0 (*middle*) and day 28 (*right*) in Fe(Salen) 10mM/10µl with AMF stimulation group. Treatment schedule was same as Supplemental Figure 2. After AMF stimulation (280 kHz and 335.4 Arms for 60 minutes), a local temperature rose to only 36.8 ℃. At 28 days after stimulation, the regression rate of brain tumor was almost equal to that of Fe(Salen) without AMF group (n=4, *p*=0.24).

(**b**) Volume-dependent changes in temperature of 10 mM Fe(Salen) solution upon exposure to AMF. Changes in the temperature of 10 mM Fe(Salen) solution are shown with an increasing volume after AMF exposure (280 kHz, 335.4 Arms) for 30 minutes. The temperature increased in a volume–dependent manner. The temperature of Fe(Salen) rose to only 36.5 ℃ at 10 µl while heat elevation to 43.0 ℃ required at least 500 µl.
